# Impact of surgical complexity and surgeon experience on phacoemulsification outcomes: a one-year retrospective study from a Saudi tertiary eye hospital

**DOI:** 10.3389/fopht.2026.1882657

**Published:** 2026-09-22

**Authors:** Rafah Fairaq, Sarah Mohammed Almuwarraee, Sarah Ayedh Almatrafi, Wejdan Saeed Alghamdi, Lojain Mohammed Maawadh, Rajiv Khandekar, Mohammed Almutlak, Anwar Ahmed, Muhammad Ali Ahad, Halah Bin Helayel

**Affiliations:** 1 Anterior Segment Division, King Khaled Eye Specialist Hospital, Riyadh, Saudi Arabia; 2 Ophthalmology Department, Prince Sultan Military Medical City, Riyadh, Saudi Arabia; 3 Ophthalmology Department, King Fahad Hospital, Al Baha, Saudi Arabia; 4 College of Medicine, Imam Abdulrahman Bin Faisal University, Dammam, Saudi Arabia; 5 Research Department, King Khaled Eye Specialist Hospital, Riyadh, Saudi Arabia

**Keywords:** audit, cataract surgery, complication, phacoemulsification, visual outcome

## Abstract

**Introduction:**

This study aimed to assess one-year outcomes of phacoemulsification cataract surgery at King Khaled Eye Specialist Hospital (KKESH), a tertiary referral teaching hospital in Saudi Arabia. Evaluated outcomes included intraoperative complications, postoperative visual outcomes, and the association of surgeon experience and surgical complexity with posterior capsule rupture (PCR) and visual outcomes.

**Methods:**

A retrospective review included 3,377 eyes that underwent phacoemulsification cataract surgery between January 1 and December 31, 2019. Data were extracted from TrakCare, the hospital’s electronic medical record system. Variables included demographics, pre- and postoperative visual acuity, intraoperative complications, combined surgical procedures, and surgeon experience. Statistical analyses were performed using Stata version 16.1. Comparisons between surgeon groups were performed using analysis of variance and chi-square tests. Multivariable logistic regression was used to identify factors associated with PCR, and multivariable linear regression was performed to evaluate predictors of postoperative visual acuity. Statistical significance was defined as P < 0.05.

**Results:**

A total of 3,377 phacoemulsification cataract surgeries were included. The mean patient age was 62.7 ± 13.1 years, 50.8% were male, and diabetes was present in 14.0% of patients. The overall posterior capsule rupture (PCR) rate was 2.8%. Although attending surgeons managed patients with poorer preoperative visual acuity and performed a greater proportion of combined procedures, surgeon level was not independently associated with PCR after adjustment for patient and surgical factors. Combined surgery was independently associated with a higher likelihood of PCR (adjusted OR 2.44, 95% CI 1.41–4.25; P = 0.002). Postoperative visual acuity improved significantly overall, while poorer postoperative visual acuity was independently associated with combined surgery, diabetes, and worse preoperative visual acuity, but not with PCR after multivariable adjustment.

**Conclusion:**

Phacoemulsification cataract surgery at a tertiary referral eye center achieved low complication rates and favorable visual outcomes, consistent with international benchmarks. After adjustment for baseline characteristics, surgical complexity rather than surgeon experience was independently associated with PCR and postoperative visual outcomes. These findings support structured case allocation in teaching hospitals and provide valuable benchmarks for surgical training and quality improvement.

## 1 Introduction

Cataract surgery is the most commonly performed ophthalmic procedure worldwide and is regarded as one of the most cost-effective healthcare interventions because of its high success rate in restoring vision and improving quality of life (1, 2). Globally, cataract remains one of the leading causes of vision impairment and blindness, with a disproportionate burden in low- and middle-income countries (3). In Saudi Arabia, cataract remained the most common cause of blindness from 1986 to 2015, with a prevalence nearly double that in high-income countries (4). With an aging population and increasing rates of diabetes, the demand for cataract surgery in the Kingdom of Saudi Arabia is expected to increase significantly in the coming years (5).

Technological advancements, such as phacoemulsification, have revolutionized cataract surgery, making it safer and more efficient. Moreover, structured training programs incorporating simulation-based learning have become integral to ophthalmic education, particularly in high-volume surgical centers (6).

Posterior capsule rupture (PCR) is one of the most significant intraoperative complications of cataract surgery and may be accompanied by vitreous loss, retained or dropped lens fragments, cystoid macular edema, retinal detachment, and poorer visual outcomes. Accordingly, risk-adjusted PCR rates are widely used as an important quality indicator and benchmark of cataract surgical performance (7, 8).

Although the international literature has extensively studied surgical outcomes and complication rates between trainees and consultants, data from the Middle East, particularly Saudi Arabia, remain limited. A few local studies have examined cataract surgery outcomes in relation to surgeon experience (9). However, direct comparisons among surgeon groups may be confounded by variations in case complexity, since more experienced surgeons often treat more challenging eyes and perform combined procedures. Adjusting for surgical complexity and baseline patient characteristics may therefore provide a more accurate evaluation of surgical performance.

A key gap in the literature is the limited evaluation of outcomes within Saudi Arabia’s tertiary eye care centers, particularly regarding how surgeons’ experiences affect surgical safety and postoperative outcomes. Existing research often lacks detailed stratification at the surgeon level and does not adequately address the effectiveness of structured training pathways in reducing complications or ensuring quality patient outcomes.

Therefore, this study aimed to evaluate intraoperative complications and postoperative visual outcomes following phacoemulsification cataract surgery at a tertiary referral eye hospital in Saudi Arabia. In addition, we examined whether surgeon experience and surgical complexity were independently associated with posterior capsule rupture and postoperative visual outcomes. These findings have significant implications for ophthalmic education and policy planning in Saudi Arabia and similar healthcare systems. Demonstrating the safety and efficacy of supervised surgical training may support the expansion of structured residency and fellowship programs, while reinforcing the value of simulation-based learning and case complexity grading in improving patient care.

## 2 Materials and methods

### 2.1 Study design

This retrospective hospital-based study was conducted at KKESH in Riyadh, Saudi Arabia. The medical records of all patients who underwent cataract surgery between January 1 and December 31, 2019, were reviewed. The study was initiated using the 2019 cataract surgery dataset, and the extensive manual extraction, cleaning, and validation of electronic medical record data contributed to the interval between data collection and completion of the analysis. The study was approved by the KKESH Institutional Review Board (IRB #RP2059) and was conducted in accordance with the principles of the Declaration of Helsinki.

### 2.2 Study population and sampling

This study included adult patients (≥18 years) who underwent cataract surgery via phacoemulsification with intraocular lens (IOL) implantation during the specified period. Patients with traumatic or pediatric cataracts, incomplete documentation, and those planned for manual extracapsular cataract extraction were excluded.

### 2.3 Data collection and management

Patient data were extracted from the hospital electronic medical record system (TrakCare). Access was limited to investigators and conducted on a password-protected computer on hospital premises.

This study did not involve any experimental procedures or additional diagnostic or therapeutic interventions beyond the standard of care.

The following variables were extracted from the electronic medical record: age, sex, diabetes mellitus status, preoperative visual acuity, surgeon level, surgical procedure, intraoperative complications, and postoperative visual acuity. Combined surgical procedures (e.g., glaucoma or vitreoretinal procedures) were also recorded and used as a surrogate measure of surgical complexity.

### 2.4 Outcomes and variables

The primary outcome was posterior capsule rupture (PCR). Secondary outcomes included other intraoperative complications and postoperative visual acuity. Postoperative visual acuity was analyzed both descriptively and using multivariable regression to identify independent predictors of visual outcome.

### 2.5 Statistical analysis

Continuous variables are presented as mean ± standard deviation (SD), and categorical variables as frequency and percentage. Baseline demographic and clinical characteristics were compared across surgeon levels (consultant, fellow, and resident) using one-way analysis of variance (ANOVA) for continuous variables and Pearson’s χ² test for categorical variables.

The primary outcome was posterior capsule rupture (PCR). The incidence of PCR and other intraoperative complications was summarized descriptively and compared between surgeon levels using Pearson’s χ² test. Multivariable logistic regression analysis was performed to identify independent predictors of PCR. Variables entered into the model included surgeon level, age, sex, diabetes mellitus, preoperative visual acuity (logMAR), and combined surgery. Results are presented as adjusted odds ratios (ORs) with 95% confidence intervals (CIs).

Postoperative visual acuity (logMAR) was compared between eyes undergoing cataract surgery alone versus combined procedures and between eyes with and without PCR using independent-samples t tests. To identify independent predictors of postoperative visual acuity, a multivariable linear regression model was constructed including PCR, combined surgery, preoperative visual acuity, age, sex, and diabetes mellitus as covariates. Regression coefficients (β) with 95% confidence intervals are reported.

All statistical analyses were performed using Stata version 16.1(StataCorp LLC, College Station, TX, USA). A two-sided P value <0.05 was considered statistically significant.

### 2.6 Ethical considerations

This study was approved by the Institutional Review Board of KKESH (RP#2059). The tenets of the Declaration of Helsinki were followed throughout the study. All collected data were anonymized to ensure participant confidentiality and privacy.

## 3 Results

### 3.1 Patient demographics

In 2019, 3,377 cataract surgical procedures were included in the analysis (**Table 1**). All surgical procedures were performed using phacoemulsification. Consultants carried out 2,041 procedures (60.4%), while fellows and residents performed 890 (26.4%) and 446 (13.2%) procedures, respectively. The mean age of the study population was 62.7 ± 13.1 years, and women accounted for 49.2% of patients. Diabetes mellitus was documented in 14.0% of eyes. Sex distribution and diabetes prevalence did not differ significantly among the surgeon groups (both P > 0.05). Patients operated on by consultants were slightly older than those treated by fellows but were similar in age to those managed by residents (P < 0.001). Eyes treated by consultants also had significantly poorer preoperative visual acuity than those managed by fellows and residents (mean logMAR 1.25 ± 0.86, 1.18 ± 0.82, and 1.04 ± 0.75, respectively; P < 0.001).

**Table 1:** Baseline characteristics by surgeon level.

Characteristic	Overall (N = 3,377)	Consultant (n = 2,041)	Fellow (n = 890)	Resident (n = 446)	P value
Age (years), mean (SD)	62.7 (13.1)	63.1 (13.4)	61.3 (13.7)	63.6 (9.5)	<0.001
Female sex, n (%)	1,663 (49.2)	1,001 (49.0)	432 (48.5)	230 (51.6)	0.56
Diabetes mellitus, n (%)	472 (14.0)	293 (14.4)	121 (13.6)	58 (13.0)	0.70
Preoperative visual acuity (logMAR), mean (SD)	1.20 (0.84)	1.25 (0.86)	1.18 (0.82)	1.04 (0.75)	<0.001

SD, standard deviation; logMAR, logarithm of the minimum angle of resolution. Data are presented as mean (standard deviation [SD]) for continuous variables and number (percentage) for categorical variables. P values were calculated using one-way analysis of variance (ANOVA) for continuous variables and Pearson’s χ² test for categorical variables.

### 3.2 Distribution of surgical complexity by surgeon level

Surgical complexity varied significantly across surgeon levels (**Table 2**). In total, 591 eyes (17.5%) underwent cataract surgery combined with another procedure during the same operative session, including glaucoma, vitreoretinal, corneal, or intravitreal injection procedures. Fellows had the highest rate of combined surgery (21.8%), followed by consultants (18.0%), while residents performed such procedures infrequently (6.5%; P < 0.001). Combined glaucoma procedures were most common among consultants (8.6%), compared with fellows (6.0%) and residents (3.6%; P < 0.001). Conversely, fellows performed the greatest proportion of combined vitreoretinal procedures (10.0%), versus consultants (6.3%) and residents (2.0%; P < 0.001). Combined corneal procedures and intravitreal injections were rare, with 17 and 21 cases, respectively.

**Table 2:** Surgical characteristics according to surgeon level.

Surgical characteristic	Overall (N = 3,377)	Consultant (n = 2,041)	Fellow (n = 890)	Resident (n = 446)	P value
Combined glaucoma surgery, n (%)	244 (7.2)	175 (8.6)	53 (6.0)	16 (3.6)	<0.001
Combined vitreoretinal surgery, n (%)	226 (6.7)	128 (6.3)	89 (10.0)	9 (2.0)	<0.001
Any combined procedure, n (%)	591 (17.5)	368 (18.0)	194 (21.8)	29 (6.5)	<0.001

Combined glaucoma surgery includes cataract surgery performed concurrently with trabeculectomy, glaucoma drainage device (tube shunt) implantation, minimally invasive glaucoma surgery (MIGS), goniosynechialysis, cyclophotocoagulation, or other glaucoma procedures. Combined vitreoretinal surgery includes cataract surgery performed concurrently with pars plana vitrectomy (PPV), silicone oil removal or exchange, epiretinal membrane peeling, macular hole surgery, retinal detachment repair, or other vitreoretinal procedures.

### 3.3 Complications

Overall, posterior capsule rupture (PCR) occurred in 96 of 3,377 eyes (2.8%). PCR was observed in 3.0% of consultant cases, 2.6% of fellow cases, and 2.5% of resident cases, with no significant difference among surgeon levels (P = 0.696). Other intraoperative complications were rare and included anterior capsule extension in 29 eyes (0.9%), zonular complications in 10 eyes (0.3%), Descemet membrane detachment in 8 eyes (0.2%), dropped lens material in 6 eyes (0.2%), and miscellaneous complications in 18 eyes (0.5%).

In multivariable logistic regression (**Table 3**) adjusting for age, sex, diabetes mellitus, preoperative visual acuity, and combined surgery, surgeon level was not independently associated with the risk of posterior capsule rupture (PCR). Compared with consultants, neither fellows (adjusted OR 0.74, 95% CI 0.40–1.35; P = 0.323) nor residents (adjusted OR 0.79, 95% CI 0.35–1.80; P = 0.582) demonstrated significantly different odds of PCR. Combined surgery was the only independent predictor of PCR, increasing the odds by more than twofold (adjusted OR 2.44, 95% CI 1.41–4.25; P = 0.002). Age, sex, diabetes mellitus, and preoperative visual acuity were not significantly associated with PCR.

**Table 3:** Multivariable logistic regression analysis of factors associated with posterior capsule rupture.

Variable	Adjusted Odds Ratio (95% CI)	P value
Surgeon level		
Consultant	Reference	—
Fellow	0.74 (0.40–1.35)	0.323
Resident	0.79 (0.35–1.80)	0.582
Age (per year)	0.98 (0.97–1.00)	0.065
Female sex	1.08 (0.66–1.78)	0.760
Diabetes mellitus	1.22 (0.63–2.40)	0.554
Preoperative visual acuity (per 1-logMAR increase)	1.10 (0.82–1.46)	0.521
**Combined surgery**	**2.44 (1.41–4.25)**	**0.002**

CI, confidence interval; logMAR, logarithm of the minimum angle of resolution. Bold values indicate statistical significance at P < 0.05.

### 3.4 Visual outcomes

Postoperative visual outcomes were significantly worse in eyes that underwent combined procedures than in those treated with cataract surgery alone (**Table 4**). The combined surgery group had poorer mean postoperative visual acuity (0.88 ± 0.80 vs 0.49 ± 0.53 logMAR; P < 0.001). However, postoperative visual acuity was not significantly different between eyes with and without posterior capsule rupture (0.69 ± 0.64 vs 0.55 ± 0.60 logMAR; P = 0.102).

**Table 4:** Postoperative visual outcomes according to combined surgery and posterior capsule rupture.

Variable	Mean postoperative logMAR (SD)	P value
Combined surgery		
No	0.49 (0.53)	
Yes	0.88 (0.80)	**<0.001**
Posterior capsule rupture		
No	0.55 (0.60)	
Yes	0.69 (0.64)	0.102

Data are presented as mean (SD). Comparisons were performed using independent-samples *t* tests. Bold values indicate statistical significance at P < 0.05.

In multivariable linear regression analysis, combined surgery remained independently associated with worse postoperative visual acuity (β = 0.33 logMAR, 95% CI 0.25–0.41; P<0.001). Worse preoperative visual acuity was also independently associated with poorer postoperative visual acuity (β = 0.37 logMAR per 1-logMAR increase, 95% CI 0.33–0.41; P<0.001). In addition, older age (β = 0.004 logMAR per year, 95% CI 0.002–0.006; P = 0.001) and diabetes mellitus (β = 0.14 logMAR, 95% CI 0.05–0.23; P = 0.001) were independently associated with worse postoperative visual acuity. After adjustment, neither posterior capsule rupture (β = −0.01 logMAR, 95% CI −0.18 to 0.16; P = 0.892) nor female sex (β = 0.001 logMAR, 95% CI −0.06 to 0.06; P = 0.986) was significantly associated with postoperative visual acuity.

## 4 Discussion

This audit of 3,377 phacoemulsification cataract procedures performed at a tertiary referral eye hospital demonstrated a low PCR rate of 2.8% and favorable postoperative visual outcomes despite the institution managing a substantial proportion of complex surgical cases. Although consultants operated on patients with poorer preoperative visual acuity and a greater proportion of combined procedures, surgeon level was not independently associated with PCR after adjustment for baseline patient characteristics and surgical complexity. Instead, combined surgery emerged as the strongest independent predictor of both PCR and poorer postoperative visual outcomes, emphasizing the importance of accounting for case complexity when evaluating surgical performance.

### 4.1 Surgical education efficiency and case allocation

The distribution of cases, with attending surgeons responsible for approximately 60% of cases and fellows and residents handling the remainder, reflects the institution’s dual role as a tertiary referral center and a surgical training hospital. Although consultants managed a higher proportion of complex combined procedures and eyes with poorer preoperative vision, complication rates remained comparable across surgeon levels. More importantly, surgeon experience was not independently associated with PCR after adjustment for surgical complexity and baseline characteristics. These findings suggest that structured case allocation and close supervision enable trainees to achieve outcomes comparable to those of experienced surgeons while maintaining patient safety. This outcome strongly contradicts studies such as that by Aaronson et al. in Finland, which highlighted disproportionately higher complication rates among less experienced surgeons (10).

This lack of disparity underscores the efficacy of the institution’s thoughtful case selection, staged independence, and robust supervision strategies, ensuring patient safety while maintaining the quality of surgical education and training. The results justify the application of a rigorous surgical education approach that includes active mentorship and simulation-based training during early learning stages. This justifies the development and implementation of case-complexity-based metrics to enhance surgical training and support patient safety.

### 4.2 Surgical complexity and combined procedures

The high proportion of combined procedures performed alongside cataract surgery reflects the hospital’s role as a high-volume referral center for complex ocular conditions. Fellows performed the greatest proportion of combined procedures overall, whereas consultants undertook the highest proportion of combined glaucoma procedures, highlighting differences in case mix across surgeon levels. More importantly, combined surgery was independently associated with a more than two-fold increase in the odds of PCR and remained the strongest predictor of poorer postoperative visual acuity.

Previous studies have highlighted the clinical and economic advantages of combined procedures in appropriately selected patients, particularly when multiple pathologies can be addressed during a single surgical session (11). Nevertheless, the increased surgical complexity observed in these eyes reinforces the importance of appropriate case selection, careful surgical planning, and risk-adjusted interpretation of complication rates when comparing outcomes across surgeons and institutions.

### 4.3 Posterior capsule rupture

Posterior capsule rupture (PCR) occurred in 2.8% of eyes, which is comparable to the 2.7% PCR rate reported in another large tertiary teaching hospital managing a high proportion of complex cataract cases. Similar to our findings, that study found no significant difference in PCR rates between consultants, fellows, and trainees, while eyes with ocular pathology had significantly higher odds of PCR (12). Our multivariable analysis likewise demonstrated that surgical complexity, represented by combined surgery, rather than surgeon level, independently predicted PCR. These findings are consistent with contemporary evidence demonstrating that risk-adjusted models incorporating ocular comorbidity, surgical complexity, and patient factors provide a more meaningful assessment of surgical performance than crude complication rates alone (8). Teaching hospitals are frequently perceived to have higher complication rates because they provide surgical training while managing more complex referral populations. However, with structured supervision, competency-based case allocation, and progressive surgical responsibility, favorable outcomes can be achieved despite this increased case complexity. This concept is supported by studies demonstrating that structured surgical training and wet-laboratory curricula substantially reduce PCR rates during resident cataract surgery (13).

### 4.4 Other intraoperative complications

Other intraoperative complications were uncommon in our cohort, including anterior capsule extension (0.9%), zonular complications (0.3%), Descemet membrane detachment (DMD; 0.2%), and dropped lens material (0.2%). These events, although infrequent, remain clinically relevant because they may increase surgical complexity and influence postoperative outcomes. Zonular dialysis has been associated with vitreous loss, PCR, and poorer postoperative visual outcomes, particularly when accompanied by additional intraoperative complications (14, 15). Similarly, dropped nucleus is a rare but potentially serious complication of phacoemulsification; a large tertiary-center series reported an incidence of 0.17%, with higher rates among resident- than faculty-performed procedures, although subsequent major complication rates were similar (16). DMD has also been evaluated in relation to surgeon experience and postoperative visual outcomes, with previous evidence suggesting that its occurrence may be influenced by surgical experience (17).

### 4.5 Postoperative visual outcomes

Postoperative visual acuity was influenced primarily by surgical complexity rather than PCR itself after adjustment for baseline characteristics. Eyes undergoing combined procedures had significantly poorer postoperative visual outcomes, likely reflecting the greater burden of pre-existing ocular pathology in this population. This observation is consistent with previous studies demonstrating that pre-existing ocular comorbidity and coexisting pathology are major determinants of visual outcomes following cataract surgery (8). Similarly, poorer preoperative visual acuity remained an independent predictor of postoperative visual outcome, emphasizing the importance of baseline ocular status when interpreting surgical success. These findings reinforce that postoperative vision reflects not only the technical success of cataract surgery but also the complexity of the underlying ocular disease.

### 4.6 Diabetes and visual outcomes

Diabetes was present in 14% of our cohort, consistent with the substantial burden of diabetes among patients undergoing cataract surgery in the region (18). Although diabetes was not independently associated with PCR, it remained an independent predictor of poorer postoperative visual acuity after adjustment for baseline characteristics. This finding likely reflects the influence of diabetes-related ocular comorbidity on visual recovery following cataract surgery and highlights the importance of optimizing systemic and ocular disease before surgery.

### 4.7 Study strengths and limitations

The strengths of this study include its large sample size, inclusion of consecutive cataract surgeries from a high-volume tertiary referral center, and the use of multivariable analyses to adjust for important clinical confounders, including surgical complexity. Nevertheless, several limitations should be acknowledged. The retrospective design limited the analysis to routinely collected electronic medical record data, and some clinically relevant variables, including standardized cataract grading, were not consistently captured in a structured format. The analysis was also limited to a single calendar year. Extensive manual extraction, cleaning, and validation of the electronic medical record data limited the feasibility of evaluating a larger multiyear cohort and contributed to the interval between data collection and completion of the analysis. Future electronic medical record systems in high-volume cataract centers should incorporate standardized documentation of surgical complexity to facilitate benchmarking, quality assurance, and clinical research. In addition, advances in artificial intelligence, particularly natural language processing and machine learning, may improve automated extraction of structured clinical information from large ophthalmic databases, facilitating larger longitudinal analyses and supporting continuous quality improvement.

In conclusion, this study demonstrates that phacoemulsification cataract surgery performed at a tertiary referral teaching hospital achieved low PCR rates and favorable visual outcomes despite managing a substantial proportion of complex surgical cases. After adjustment for baseline patient characteristics, surgical complexity rather than surgeon experience was independently associated with PCR and postoperative visual outcomes. These findings support structured case allocation, competency-based surgical training, and continued risk-adjusted quality assurance in high-volume teaching centers while providing contemporary benchmark data for cataract surgery in Saudi Arabia.

## Data Availability

The de-identified data supporting the findings of this study are available from the corresponding author upon reasonable request, subject to institutional policies and ethical approval.
